# Blood pressure lowering and potassium intake

**DOI:** 10.1038/s41371-020-00396-1

**Published:** 2020-08-12

**Authors:** Biff F. Palmer, Deborah J. Clegg

**Affiliations:** 1 Department of Medicine, Division of Nephrology, University of Texas Southwestern Medical Center, Dallas, TX USA; 2 College of Nursing and Health Professions, Drexel University, Philadelphia, PA USA

**Keywords:** Health care, Prognosis

In this issue Chaudhary et al., accessed the Dietary Approaches to Stop Hypertension Sodium Trial (DASH-Sodium) dataset via the NHLBI and BioLINCC to obtain data on systolic blood pressure (SBP) from individuals deemed to be salt sensitive (SS) or salt resistant (SR) based on the response to changes in Na^+^ intake [1]. Importantly, Na^+^ intake in the trial ranged from 1.5 to 3.3 g/day over a 30-day period and participants were also encouraged to increase K^+^ intake [2]. The authors examined the relationship between SBP and urinary Na^+^ and K^+^ excretion, and the Na^+^/K^+^ ratio via linear regression. Consistent with previous literature, there was a significant drop in SBP in both the SS and SR participants assigned to the DASH-low sodium diet as compared to baseline screening values. However, the authors indicated they found no association between urinary K^+^ excretion or the urinary Na^+^/K^+^ ratio and change in SBP in either the SS or SR groups. This lack of association occurred even though urinary K^+^ excretion increased from 2.2 to 3.2 and 2.1 to 3.2 gm/d on the DASH-low sodium diet when compared to pre-screening levels suggestive of an increase in dietary consumption of K^+^. Furthermore, the urine Na^+^/K^+^ ratio decreased from 1.9 to 0.6 and 1.8 to 0.5 in the SR and SS groups respectively. The authors concluded that despite the perceived increase in K^+^ consumption and reduction in Na^+^ consumption, the Na^+^/K^+^ ratio bore no relationship to SBP.

Potassium (K^+^) is an extremely important mineral, as supported by National Academy of Science, Engineering, and Medicine Dietary Reference Intakes (DRI), and has been designated as a “nutrient of public health concern” due to its general *under* consumption [3]. In 2004, the Food and Nutrition Board of the Institute of Medicine established recommended intake levels of 4700 mg/day of K^+^ [4]. Despite these recommendations, data from National Health and Nutrition Examination Survey (NHANES) 2007–2008 estimated mean intakes in the United States to be 2290 mg/day for women and 3026 mg/day for men, substantially lower than the suggested values [5]. This relative “deficiency” is even more noteworthy when one considers the K^+^ intake of prehistoric man was estimated to be 15,000 mg/day, which actually exceeds the NHANES recommendations by a factor greater than 4 [6]. Despite studies demonstrating blood pressure lowering effects of increasing K^+^ intake, the 2019 DRI concluded more evidence is required to support a DRI for K^+^ [7].

The daily intake of Na^+^ in Western industrialized societies is about three times higher than the daily intake of K^+^ on a molar basis [7]. Urinary excretion of K^+^ and Na^+^ are the most accurate measure of dietary consumption [8]. Data suggest the urinary Na^+^/K^+^ ratio has a stronger association with blood pressure than Na^+^ or K^+^ independently [9, 10]. A urinary Na^+^/K^+^ ratio <1 has been recommended as a beneficial target to improve long term blood pressure control [11, 12]. There are two ways of achieving this - either through dietary restriction of Na^+^ and/or through significant increases in dietary K^+^. While Chaudhary et al. conclude a urinary K^+^ excretion >1 g/day and a reduction in the urinary Na^+^/K^+^ ratio are not associated with lower SBP, the observation that SBP is increased when intake is <1 g K^+^/day suggests low dietary intake of K^+^ is associated with HIGHER blood pressure and supports the argument for increased dietary K^+^ intake. The findings of this study should be interpreted in the context of numerous other studies demonstrating increased K^+^ intake given as a supplement or achieved by a diet enriched in fruits and vegetable lowers BP in hypertensive and normotensive individuals [13–15].

Mechanistically, increased K^+^ intake leads to accumulation in the interstitium of the kidney causing an inhibitory effect on the thick ascending limb and to a lesser extent proximal tubular NaCl reabsorption resulting in increased flow and Na^+^ delivery to the distal nephron [16]. In addition, slight increases in plasma K^+^ concertation are sensed by cells in the initial portion of the distal convoluted tubule leading to an inhibitory effect on the thiazide sensitive Na^+^-Cl^−^ co-transporter (NCC). Increased flow and Na^+^ delivery to the aldosterone sensitive distal nephron (ASDN) stimulates electrogenic and flow mediated K^+^ secretion. In addition to maintaining a normal plasma K^+^ concentration, these natriuretic effects contribute to the blood pressure lowering effect of high K^+^ intake. By contrast, reductions in K^+^ intake lead to increased activity of NCC and limit K^+^ secretion by reducing Na^+^ delivery and flow to the ASDN. The effect of a K^+^ deficient diet to reduce K^+^ secretion at the expense of increased Na^+^ retention has been linked to the pathogenesis of salt-sensitive hypertension.

The blood pressure lowering effect of increased K^+^ intake is more pronounced in individuals with high salt intake. This effect is important since the population studied by Chaudhary were consuming a relatively low Na^+^ diet. In this setting, a small increase in dietary K^+^ intake, insufficient to cause a reduction in blood pressure, would cause a large decrease in the urinary Na^+^/K^+^ ratio. As a result, there would be no correlation between the ratio and blood pressure lowering. In this regard, the dietary intervention in the trial was to increase K^+^ intake to 4.7 gm/d and yet the majority of participants in the analysis had intakes in the range of 1–2 gm/d based on urinary K^+^ measurements. We believe current guidelines recommending increasing K^+^ intake for the prevention and treatment of hypertension are justified [17].
